# Influence of Post Processing and Alloying with Zn on Formability of an Mg–2Al–0.5Ca–0.4Mn Alloy

**DOI:** 10.3390/ma19153168

**Published:** 2026-07-24

**Authors:** Christopher Hale, Zhigang Xu, Prithu Dhar, Jagannathan Sankar

**Affiliations:** Center for Advanced Materials and Smart Structures (CAMSS), Department of Mechanical Engineering, North Carolina A&T State University, Greensboro, NC 27411, USA; pdhar1@aggies.ncat.edu (P.D.); sankar@ncat.edu (J.S.)

**Keywords:** magnesium alloys, differential speed rolling, static recrystallization, post annealing, texture weakening, formability

## Abstract

Single-pass differential speed rolling (DSR) is an effective route for strengthening magnesium alloys through grain refinement induced by dynamic recrystallization (DRX); however, the accompanying strong basal texture often limits ductility and formability. In previous work, an Mg–2Al–0.5Ca–0.4Mn alloy (namely AXM20504) in the T4 condition was subjected to single-pass DSR with thickness reductions of 20% and 40% with a preheat temperature of 400 °C and a roll temperature of 300 °C, followed by post-annealing at 350–450 °C for durations of 20–60 min to systematically investigate static recrystallization (SRX), texture evolution, and mechanical response. Electron backscatter diffraction (EBSD) revealed that post-annealing promoted progressive SRX, with nearly complete recrystallization achieved at 450 °C for 40 min. This transition was accompanied by substantial basal texture weakening, reduced kernel average misorientation (KAM), and significantly lower grain orientation spread (GOS), indicating effective stress relief and formation of strain-free grains. As a result, tensile ductility increased from ~5% in the 40% as-rolled condition to ~12% after optimized post-annealing, while ultimate tensile strengths were retained above 200 MPa, much higher than the initial T4 strength. While these findings demonstrate that post-annealing is a critical step in restoring ductility and enhancing the formability of DSR-processed Mg alloys, certain types of alloying can also assist in a favorable balance between strength and formability for sheet forming applications. Alloying with Zn has been shown to improve ductility to a higher than 20% elongation at break as compared to 5% for the T4 AXM base material, showing that processing techniques and alloying have a high impact on the formability of Mg-based alloys. The zinc-based alloys in this study include Mg-2Al-0.5Ca-0.4Mn-0.5Zn (namely AXMZ2050405) and Mg-2Al-0.5Ca-0.4Mn-1Zn (namely AXMZ205041), both of which demonstrated improvements in mechanical properties as compared to the base alloy, AXM20504.

## 1. Introduction

Magnesium alloys offer significant potential for lightweight structural applications due to their low density and high specific strength [1]. Nevertheless, their widespread use in sheet-metal forming remains constrained by limited room temperature formability, which arises from the hexagonal close-packed crystal structure and the restricted activity of non-basal slip systems [2]. Processing strategies that promote grain refinement and texture modification are therefore critical for improving ductility while maintaining strength [3]. In addition, other techniques such as alloying with zinc can be equally important in the improvement of mechanical properties enhancing the formability and performance of the alloy [4,5,6].

Differential speed rolling (DSR) introduces shear deformation through asymmetric roll speeds, promoting microstructural refinement and strengthening via dynamic recrystallization, as well as basal pole tilting. While numerous studies have demonstrated the effectiveness of DSR in improving strength, the resulting strong basal texture frequently suppresses ductility [7,8]. In contrast to dynamic recrystallization, static recrystallization during post-annealing provides an opportunity to relieve internal stresses, reorient grains, and weaken texture inheritance from rolling, thus improving ductility [9,10,11,12,13,14].

Magnesium is alloyed with elements that improve mechanical properties, which is why Ca and Zn seem to be some of the most suitable elements as demonstrated in previous work [15,16,17,18,19]. These elements are seemingly preferred since zinc is added to improve strength at ambient temperature, elongation and corrosion resistance, while Ca has been added to reduce oxidation during heat treatment and also to improve strength [19,20]. A low Ca content refines grain size and weakens the Mg texture. Also, alloying by calcium enables the solid solution and precipitate strengthening and refinement of the structure. It has been shown that the strength of the alloy and the amount of secondary phases increases with increasing calcium content, but ductility decreases. Simultaneous addition of calcium and zinc to magnesium leads to the formation of the ternary phase Ca_2_Mg_6_Zn_3_, which could increase high-temperature properties. Nevertheless, results show that alloying with a low amount of both zinc and calcium is preferred [19].

AXM magnesium alloys (Mg-Al-Ca-Mn) have been shown to generally exhibit finer, more stable grain sizes after rolling and post-annealing compared to AXMZ (Mg-Al-Ca-Mn-Zn) alloys, which is primarily due to the higher efficiency of Al_2_Ca precipitates in restricting grain boundary mobility during recrystallization [19,20]. Thus, the AXM maintains a smaller average grain size due to the higher amounts of the precipitate in contrast to the precipitates in the Zn-containing AXMZ. While it has been shown that rolling refines both alloys, the AXM’s Al_2_Ca phase has shown stronger Zener pinning, while the zinc in AXMZ can increase grain boundary mobility. With the addition of Zn leading to higher grain boundary mobility, this leads to better ductility as exhibited by the AXMZ.

Previous work on Mg–Al–Ca–Mn alloys has shown that Ca additions enhance high-temperature strength but can further degrade ductility after rolling. Among AXM alloys, Mg–2Al–0.5Ca–0.4Mn exhibits a promising balance between mechanical performance and processability [7]. Building upon earlier studies of DRX originating from DSR, the present work focuses on the role of alloying with Zn in addition to the DSR processing with post-annealing that activates SRX to restore ductility while maintaining strength [9]. The advantages imparted by DSR with post-annealing are enhanced by alloying with Zn, significantly improving ductility and formability at room temperature while retaining mechanical strength. The AXM alloys are work that was completed in previous studies while the addition of the zinc for the AXMZ alloys is represented in the current study.

## 2. Materials and Methods

### 2.1. Materials Preparation

As in our previous work with AXM alloys, the casting process used a box-melting furnace and a preheated permanent steel mold in an argon-filled glove box [7,8]. PANDAT software Version 2020 (CompuTherm LLC, Middleton, WI 53562, USA) was used to determine the composition [21]. Based on a previous study, Mg-2Al-0.5Ca-0.4Mn (also referred to as AXM20504) was the chosen composition for the ingot prepared for the rolling and post-annealing study [7,9]. In addition, 0.5 and 1 wt.% zinc was added to create the AXMZ alloys (AXMZ2050405 and AXMZ205041) to examine the effect of alloying with Zn. After a T4 heat treatment at 500 °C for 30 h, the T4-treated alloy ingot with a size of 60 mm × 28 mm × 104 mm was cut into rectangular plates with dimensions of 65 mm long, 24 mm wide, and a thickness of 4 mm by wire EDM (Sodick VL400Q, Sodick, Schaumburg, IL, USA) and prepared for differential speed rolling.

### 2.2. Rolling

The plates mentioned above were chamfered on each edge to reduce the potential for side cracking and labeled for rolling at 40% reduction—similar to the reductions chosen in previous work [7,9]. The 40% reduction represented a more complete DRX and SRX as demonstrated in previous work [9]. The mill speed was set to 4 m/min for the top and 2 m/min for the bottom rolls, and boron nitride lubricant was applied to the surface of the rolls prior to rolling to prevent the material from sticking to the surface of the rolls. Also, the roll temperature of the mill was maintained at 300 °C for both the AXM and AXMZ alloys to assist in maintaining the pre-heat temperature of the specimens all preheated to 400 °C for 10 min prior to rolling.

### 2.3. Post-Annealing

Post-annealing on the 40% rolled AXM and AXMZ samples was performed in a box furnace, using a post-annealing temperature of 450 °C for 40 min based on previous studies that have shown that this duration and temperature are the most effective in reaching complete recrystallization as part of the SRX process [9]. Samples were then removed from the furnace and quenched in water to retain the microstructure.

### 2.4. Microstructural Characterization

Both the as-rolled and post-annealed AXM and AXMZ alloys were prepared for SEM/EBSD metallography using standard mechanical grinding SiC papers and final polishing, using 0.05 μm Al_2_O_3_ suspension to the mid-section of the sample thickness. The samples were then subjected to electrochemical polishing on a Struers TenuPol-5 following a standard electrolytic preparation procedure to achieve a deformation-free surface for metallographic analysis. They were also subjected to ion milling (Fischione Instruments, Model 1061, Export, PA, USA) to prepare for scanning electron microscopy (SEM) (SU8000, Hitachi, Chiyoda City, Tokyo, Japan) and electron backscatter diffraction (EBSD). Ion milling was performed for 2 h at 4 kV, followed by another 2 h at 2 kV, with a 2° tilt for both steps. For the AXM and AXMZ alloys, microstructural features were examined on the normal direction (ND) plane. Crystallographic information was obtained via EBSD (Symmetry, Oxford Instruments, Abingdon, UK).

Scanning Transmission Electron Microscopy (STEM) specimens were prepared by polishing to about 100 µm mechanically, followed by electrochemical polishing until a perforation is acquired. They were further ion milled (Fischione Model 1010). STEM analysis was done on the specimens using the STEM function of the SU8000 SEM at 30 kV.

The crystallographic information acquired by EBSD allowed for the generation of inverse pole figure (IPF) maps and pole figures, facilitating the examination of the quantity and grain size of dynamically recrystallized (DRX) grains and the pole figure intensity and orientation of the grains. SEM and EBSD images were captured at various magnifications, for close examination of microstructural features taken from the mid-plane of the DSR processed sample. The EBSD scanning utilized the acquisition parameters of 15 kV and 15 μA. Aztec Version 6.0 and Aztec Crystal Version 2.2 software played a vital role in conducting various measurements and analyses, including DRX grain measurements, area fraction measurements, pole figures, tilt angles, and low/high grain-boundary angles. The integration of EBSD contributed to a comprehensive understanding of the microstructural characteristics, offering insights into the amount, size, and distribution of DRXed grains and the orientation of grains and associated pole intensity.

### 2.5. Mechanical Testing

Three tensile specimens were prepared according to ASTM E8/ASTM E8M [22], using a wire-EDM for the T4, as-rolled, and the annealed samples of AXM and AXMZ. The tensile direction was parallel to the rolling direction. Dimensions at the gauge were 15 mm in length, 3.0 mm in width, and 2.4 mm thick for the 40% reduction plates. All the samples were tested using the Instron Model 3000 machine (Instron, Norwood, MA, USA) at an estimated strain rate of 1.1 × 10^−3^ s^−1^. The data was recorded for each test in an Excel file for further analysis. Calculations for the stress, strain, and elongation were determined from the stress–strain curves and the strain hardening exponent calculations were determined using the Hollomon equation that extracts the *n*-value from the stress–stain data obtained from the tensile test wherein the stress–strain data is plotted on a log–log curve and the resulting straight line is the *n*-value is determined from the slope of the curve.

## 3. Results

### 3.1. Microstructural Evolution

Single-pass DSR resulted in progressive grain refinement with increasing thickness reduction, as evidenced by EBSD inverse pole figure maps. Post-annealing activated static recrystallization, with the extent of SRX dependent on both temperature and duration. Based on previous work, the temperature and duration of 450 °C at 40 min showed nearly 100% recrystallization with moderate grain growth at 60 min [9]. Thus, a comparison between the AXM20504 and AXMZ alloys was made where both the AXM and AXMZ were post-annealed at 450 °C for a duration of 40 min.

**Figure 1:**
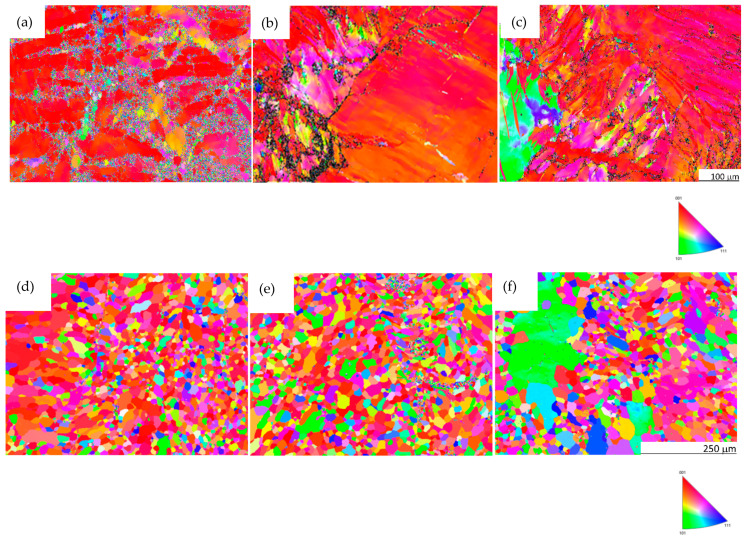
(**Top**) EBSD IPF maps for DSR 4:2 40% showing as-rolled for (**a**) AXM20504, (**b**) AXMZ2050405, and (**c**) AXMZ205041 at a magnification of 500× and (**Bottom**) EBSD IPF maps for DSR 4:2 40% that were post-annealed at 450 °C and 40 min for (**d**) AXM20504, (**e**) AXMZ2050405, and (**f**) AXMZ205041 at a magnification of 200×.

EBSD inverse pole figure (IPF) maps illustrating the DRX grain structures after rolling at a 4:2 ratio and 40% reduction are presented in Figure 1a–c for AXM20504, AXMZ2050405, and AXMZ205041, respectively, with a magnification of 500× to clearly show the DRX grains throughout the material. These are contrasted with the corresponding microstructures after post-annealing at 450 °C, shown in Figure 1d–f, which exhibit characteristic features of static recrystallization (SRX) and all at a magnification of 200× to clearly show the recrystallized grains throughout the specimens. As observed in Figure 1d–f, fully recrystallized grain structures are evident following annealing. For the rolled and post-annealed specimens, the addition of Zn showed a slight refinement of average grain size as seen in Table 1.

**Figure 2:**
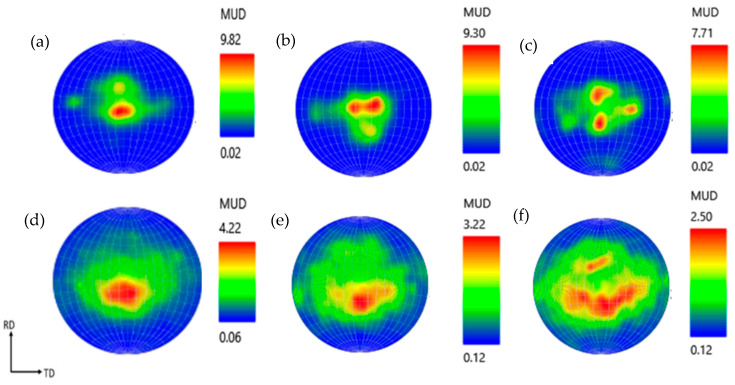
EBSD pole figures for DSR 4:2 40% as-rolled showing (**a**) AXM20504, (**b**) AXMZ2050405, and (**c**) AXMZ205041 and the annealed 450 °C, 40 min for (**d**) AXM20504, (**e**) AXMZ2050405, and (**f**) AXMZ205041.

Pole figure analysis presented in Figure 2 indicates a pronounced spreading and overall weakening of the basal texture after annealing. Specifically, the maximum texture intensity in the annealed specimens decreases by more than 50% relative to the rolled condition. This reduction in texture strength is attributed to the nucleation and growth of strain-free grains with more randomized crystallographic orientations during static recrystallization (SRX) [19,20]. 

**Figure 3:**
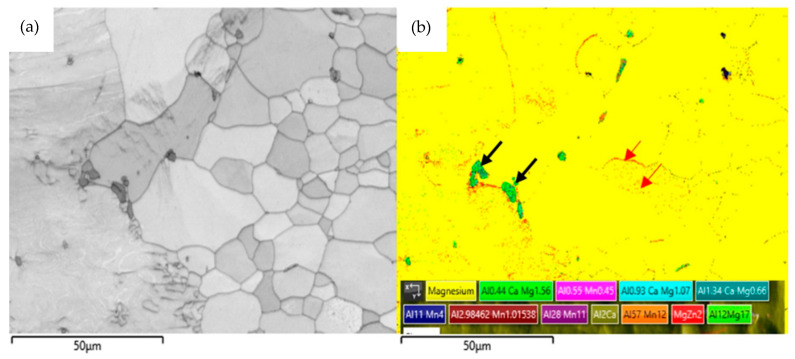
(**a**) Band contrast image of the AXMZ205041 and (**b**) associated EBSD phases for the AXMZ205041 annealed at 450 °C and 40 min showing secondary phases present both within the matrix and around the grain boundaries denoted by red arrow (MgZn_2_) and primarily around grain boundary denoted by black arrow (Al_0.44_CaMg_1.56_), also commonly known as (Mg,Al)_2_Ca.

The secondary phases present in AXMZ205041, both within the α-Mg matrix and along grain boundaries, are shown in Figure 3. As indicated by the red and black arrows, two dominant intermetallic phases are observed: MgZn_2_ (red arrows) and Al_0.44_CaMg_1.56_ (black arrows). Following rolling and post-annealing, the microstructure exhibits a bimodal grain size distribution. Figure 3a shows the band contrast image, where a mixture of coarse, fully recrystallized grains and localized clusters of finer grains and precipitates is evident. Figure 3b presents the corresponding phase map, highlighting the spatial distribution of intermetallic phases, particularly along grain boundaries and in localized regions of the microstructure.

In Figure 3a, the grains are on the order of tens of microns and have an average grain size of 30.95 ± 12.3 μm. The relatively smooth grain morphology indicates that static recrystallization has largely been completed, followed by grain growth. This observation is consistent with a reduced dislocation density associated with a recrystallized microstructure. The larger grains are interpreted as having grown during annealing, driven by the reduction in stored deformation energy and facilitated by grain boundary migration. In contrast, the finer grains appear clustered and exhibit slightly irregular morphologies, suggesting spatial variations in recrystallization kinetics. In some regions, grain growth appears to be inhibited, indicating localized Zener pinning by second-phase particles. These observations point to heterogeneous recrystallization behavior, likely governed by the local distribution of solute elements and precipitates in the AXMZ alloy. The phase map in Figure 3b shows the α-Mg matrix (yellow) along with secondary intermetallic phases enriched in Ca, Mn, and Zn, primarily MgZn_2_ and Al_0.44_CaMg_1.56_. The coarse Al_0.44_CaMg_1.56_ particles (black arrows), also commonly known as (Mg,Al)_2_Ca, are concentrated in localized regions and are likely remnants of deformation-segregated or partially undissolved intermetallics from the rolled condition. These coarse particles can act as nucleation sites for recrystallization, promoting the formation of fine recrystallized grains via particle-stimulated nucleation (PSN). The finer MgZn_2_ precipitates (red arrows) are dispersed along grain boundaries and within the grain interiors, forming a discontinuous network within the Mg matrix.

The presence of fine precipitates decorating grain boundaries around larger grains suggests a Zener pinning effect, which impedes grain boundary migration and helps stabilize grain size, although it appears insufficient to completely suppress grain coarsening [19,20]. Regions with a lower density of precipitates within larger grains allow for more extensive grain growth. Overall, the observed microstructure indicates that the annealing conditions were sufficient to promote recrystallization, while the non-uniform spatial distribution of second-phase particles resulted in localized grain refinement alongside regions of enhanced grain growth. Consequently, the microstructure comprises predominantly large, equiaxed recrystallized grains interspersed with finer, particle-stabilized regions.

### 3.2. Internal Stress Relaxation/Recovery and Recrystallization Metrics

In a previous study, both metrics decreased substantially with increasing annealing temperature, indicating effective removal of stored deformation energy [9]. The lowest KAM and GOS values were observed after annealing at 450 °C for 40 min, confirming extensive stress relief and the formation of recrystallized grains indicative of static recrystallization [9]. Below are the KAM and GOS graphs comparing the as-rolled and annealed samples at 450 °C for 40 min for the single-pass rolled samples for AXM20504, AXMZ2050405, and AXMZ205041. Table 2 below also shows the KAM and GOS values for each of the rolled and annealed sample conditions.

While the GOS for the AZMZ205041 is near 1, the specimen at 40% reduction is not near complete recrystallization in the as-rolled state, which is evident by viewing the IPF map that clearly shows areas of large and small grains. Furthermore, in some cases, a GOS less than 1 does not always imply near-complete recrystallization since a map will generally show a range of 85% to 95% of grains being very small and fine grains. Even so, the post-annealed AXMZ205041 had a GOS of half that of the “as-rolled”, indicating that the AXMZ alloy became further recrystallized by post-annealing and even more so relative to its AXM20504 and AXM2050405 counterparts. While the KAM map for the AXMZ205041 is predominantly blue for the “as-rolled” and annealed conditions, the KAM value for the annealed is reportedly half of the “as-rolled” state, wherein it is visibly bluer, representing the lower KAM value reported.

**Figure 4:**
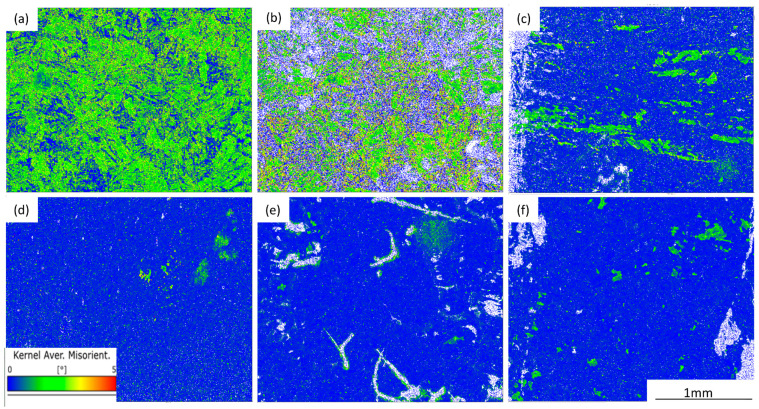
Kernel average misorientation (KAM) of the as-rolled for (**a**) AXM20504, (**b**) AXMZ2050405, and (**c**) AXMZ205041 alloys following DSR 4:2 at 40% reduction with preheat of 400 °C and rolling temperature of 300 °C compared to the post-annealed at 450 °C and 40 min for (**d**) AXM20504, (**e**) AXMZ2050405, and (**f**) AXMZ205041.

**Figure 5:**
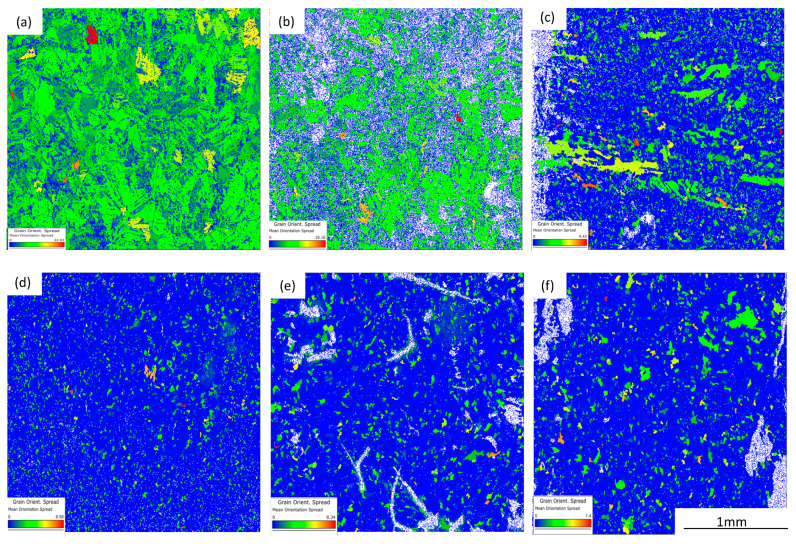
Grain orientation spread (GOS) of the as-rolled (**a**) AXM20504, (**b**) AXMZ2050405, and (**c**) AXMZ205041 alloys following DSR 4:2 at 40% reduction with preheat of 400 °C and rolling temperature of 300 °C compared to the post-annealed condition for the corresponding materials in (**d**–**f**).

Figure 4 and Figure 5 show both the KAM and GOS generally decreased with annealing, which was due to effectively removing the stored deformation energy and reducing lattice distortion with formation of newly recrystallized grains. Figure 4 shows the KAM for the rolled and annealed AXM and AXMZ alloys showing how the KAM decreased for both alloys with annealing. On the other hand, Figure 5 shows how the GOS likewise decreased for both alloys with subsequent annealing.

### 3.3. STEM Analysis

**Figure 6:**
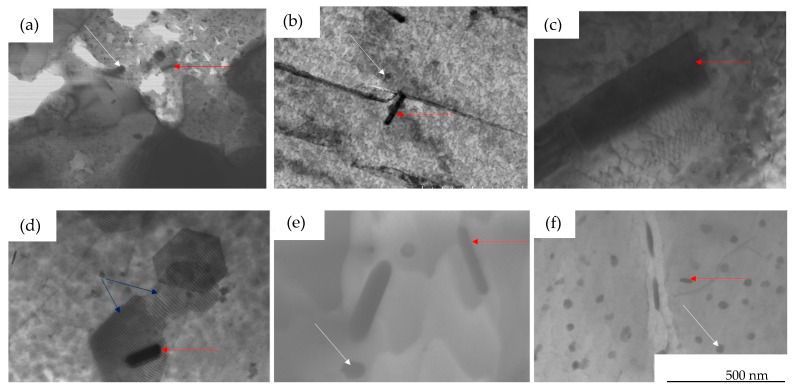
STEM analysis of the as-rolled (**a**) AXM20504, (**b**) AXMZ2050405, and (**c**) AXMZ205041 alloys following DSR 4:2 at 400 °C at 40% compared to the post-annealed condition (**d**) AXM20504, (**e**) AXMZ2050405, and (**f**) AXMZ205041.

Morphology-based STEM micrographs of the single-pass rolled condition are depicted in Figure 6a–c, and the annealed condition at 450 °C and 40 min in Figure 6d–f. While TEM is often preferred to STEM due to superior field of view, acquisition speed, and depth of field, STEM can provide superior localized analytical capability. In the as-rolled condition, rod-like features tend to appear finer and less uniform. For the annealed condition, the precipitates, including the rod precipitates, tend to be more well-defined. The AXMZ alloys with zinc appear to correlate to an increased number of discrete precipitates as seen in Figure 6e,f with a more clearly defined rod morphology. The background of the micrographs shows strong deformation contrast that appears consistent with high dislocation density. The large, faceted plate-like structure seen in Figure 6d appears to show internal parallel fringes (strain contract or thickness fringes) with reduced deformation contrast compared to the rolled state. Overall, the Zn addition promotes the formation, refinement, and stabilization of rod-shaped precipitates which evolve from diffuse, defect-associated features in the rolled state to more well-defined rods after annealing [23,24]. Also, increasing the Zn content appears to drive rod coarsening and increase precipitate density while also introducing secondary population of fine, equiaxed particles [23,24]. In contrast, the Zn-free alloy shows more limited rod development with precipitation favoring larger, faceted particles rather than the uniform rod dispersion. The red arrows highlight more of the rod-like precipitates while the white arrow highlights more of the sphere-like precipitates, and the blue arrow highlights some larger faceted precipitates.

### 3.4. Fractography of the Tensile Tested Specimens

**Figure 7:**
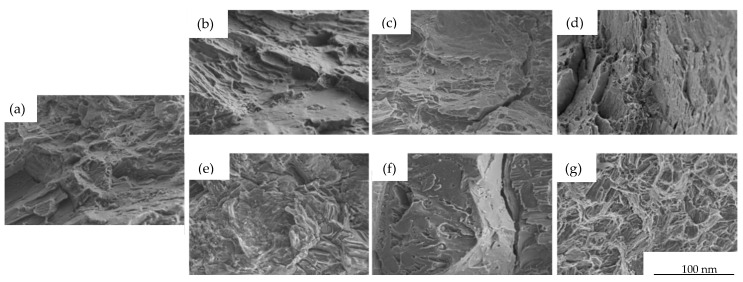
SEM Fractography of (**a**) AXM20504 (T4) and as-rolled DSR 4:2 (40%) for (**b**) AXM20504, (**c**) AXMZ2050405 (**d**) AXMZ205041 and post-annealed at 450 °C and 40 min for (**e**) AXM20504 (**f**) AXMZ2050405 and (**g**) AXMZ205041.

SEM fractography revealed a highly rough and torn fracture surface characterized by extensive plastic deformation and step-like tearing features in the T4 sample seen in Figure 7a. The fracture morphology showed fibrous ligaments and interfacial separation that is consistent with microvoid coalescence under a shear-dominated loading. The absence of cleavage facets suggests a predominantly ductile failure mechanism that showed a mixed-mode contribution [25]. In contrast to the T4, the AXM20504 DSR 40% image in Figure 7b shows elongated, shear-aligned ridges with torn ligaments also representative of a ductile, shear-dominated fracture from the final overload. Likewise, the AXMZ2050405 DSR 40% in Figure 7c and AXMZ205041 in Figure 7d show layered sheet-like features with tear ridges and shear steps with limited flat facets or grain-boundary faceting. On the other hand, the annealed AXM20504 at 450 °C and 40 min in Figure 7e demonstrates a faceted lamellar fracture surface with river patterns. In contrast, the AXM205041 DSR 40% annealed at 450 °C for 40 min in Figure 7g shows a more dimpled morphology, though the dimples are irregular and elongated with almost no river patterns or cleavage steps suggesting microvoid nucleation, growth, and coalescence, which is the hallmark of ductile fracture. Also, the elongated non-equiaxed dimples suggest a shear-dominated or mixed-mode loading. This analysis is consistent with other research showing how there is a transition from a predominantly ductile failure in magnesium to a mixed-mode (ductile and brittle) fracture in the calcium-alloyed specimens (AXM) [25].

Overall, the fractography indicates that most of the features of the as-rolled specimens are characteristic of ductile and brittle fracture in the AXM (due to higher Ca) with a more ductile fracture by adding Zn, since the Zn produces coarse secondary particles and fine, deep dimples, allowing for enhanced ductile fracture behavior and improved stretch formability [19,20]. The dimples, however, were more evident in the annealed specimens, which indicate that microvoid nucleation, growth, and coalescence (ductile fracture) support material stretching in the form of elongated non-equiaxed dimples prior to fracture.

### 3.5. Mechanical Properties and Formability

**Figure 8:**
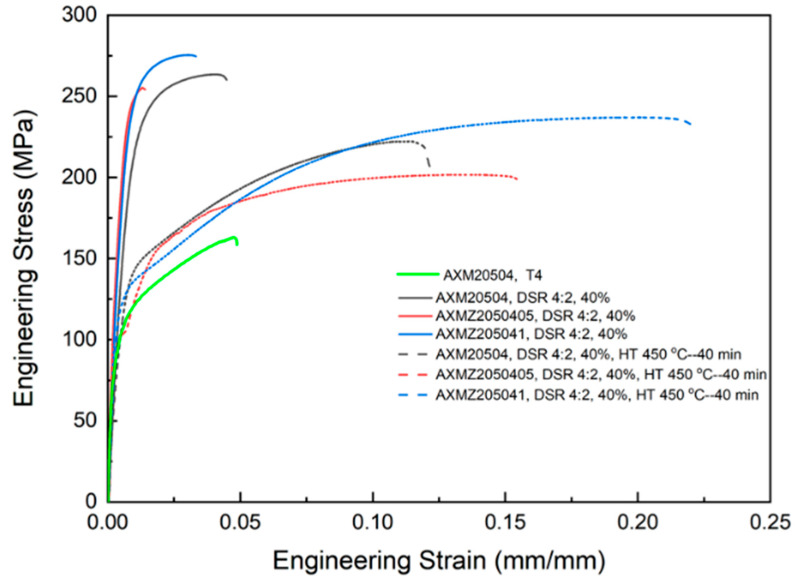
Stress–strain curves for the initial T4 AXM20504 compared to the rolled specimens at DSR 4:2 (40%) and the post-annealed condition at heat treatment of 450 °C and 40 min.

The goal of the differential speed rolling was to produce fine DRX grains throughout the microstructure, which led to significant strengthening of the alloy through grain size refinement. The follow-up post-annealing relaxed the lattice structure, relieved internal stresses, as well as formed new grains by SRX with slightly increasing the average grain size, which produced a pronounced increase in tensile ductility, particularly for the DSR 40% specimens. While rolling imparted high strength to nearly 260 MPa for the base AXM20504 alloy at the expense of elongation, subsequent annealing enabled elongation to go from 5% to approaching 12% while maintaining tensile strengths well above 200 MPa, as seen in Figure 8. The calculated strain hardening exponent went from 0.18 for the annealed AXM20504 to 0.225 for the annealed AXMZ205041, with zinc reflecting an improvement in uniform deformation and enhanced formability.

The combined effects of grain size normalization, texture weakening, and activation of non-basal slip systems contribute to the enhanced mechanical performance observed after rolling and subsequent post-annealing. These findings emphasize the importance of integrating post-annealing with DSR processing to mitigate the inherent strength–ductility tradeoff in Mg alloys. Figure 8 presents the engineering stress–strain behavior for: (a) DSR (4:2) as-rolled AXM20504, AXMZ2050405, and AXMZ205041 alloys, and (b) their corresponding post-annealed conditions. The annealed alloys exhibit substantially higher strain-to-failure and improved ductility compared to their rolled counterparts. Furthermore, alloying with 0.5 and 1.0 wt% Zn results in a pronounced increase in ductility, as evidenced by the AXMZ2050405 and AXMZ205041 compositions relative to the base AXM20504 alloy (Table 3).

Although post-annealing leads to a reduction in strength compared to the rolled condition, the overall mechanical response remains improved. Specifically, the Zn-containing alloys demonstrate a favorable balance of strength and ductility, outperforming the base AXM20504 alloy in the T4 condition.

## 4. Discussion

Differential speed rolling (DSR) coupled with post-annealing has been shown to enhance the mechanical properties and formability in Mg-based alloys. Through DSR, grain refinement is achieved, which improves the mechanical strength through significant grain refinement while post-annealing reduces the lattice strain/internal stresses and generates new grains through SRX, leading to the texture weakening and improvement in ductility as seen in Figure 1 and Figure 2. The addition of Zn to AXM (Mg-Al-Ca-Mn) alloys has been shown by previous studies to promote the formation of complex precipitates (Ca_2_Mg_6_Zn_3_) that can lead to increased strength through precipitation hardening and improved ductility by facilitating dynamic recrystallization during processing [19].

The Zn addition, specifically in small amounts of 0.6 to 1.0 wt%, has been shown to refine grain size in the AXMZ alloys by inhibiting grain growth through segregation of the Zn atoms at grain boundaries and particles, as seen in Figure 3, which encourages dynamic recrystallization (DRX) [19,20]. The Zn in the alloy reacts to form ternary or quaternary precipitates (Mg-Al-Ca-Zn or similar), which can also serve as nucleation sites for new, small grains, contributing to a particle-simulated nucleation (PSN). The precipitates observed in the AXMZ alloys were primarily (Mg,Al)_2_Ca and MgZn_2_. EBSD phase analysis indicated that the precipitates were consistent with Al_2_Ca and MgZn_2_ phases. Although the indexing results strongly suggest these phase assignments, additional compositional confirmation using EDS or crystallographic verification using SAED was not available in the present study. Furthermore, alloy composition verification through OES or ICP-OES is not reported to detect small deviations in Zn due to limitations in this capability.

In this study, the addition of Zn is shown to modify the typical basal texture which encourages “texture weakening” and shifting the basal pole away from the normal direction of the specimen toward the transverse direction as seen in Figure 2. In contrast, AXM alloys are known to retain relatively fine grain structures after thermomechanical processing due to enhanced Zener pinning from the precipitates. However, the addition of Zn in AXMZ alloys promotes increased grain boundary mobility, leading to accelerated grain growth and more pronounced grain coarsening during post-annealing. This increased mobility can be beneficial, contributing to improved ductility and formability. At the same time, Zn additions enhance the precipitation behavior by refining and increasing the dispersion of Al_2_Ca particles, thereby increasing the overall volume fraction of intermetallic phases. Zn promotes the formation of MgZn_2_ precipitates, which preferentially decorate grain boundaries and contribute to strengthening of the Mg alloy. In contrast, Zn also facilitates the activation of non-basal slip systems, thereby improving ductility and stretch formability [26,27].

Another factor to consider regarding texture weakening is the evidence of non-basal slip activity with both the activation of prismatic and pyramidal slip systems. Table 4 below shows the Schmid factor analysis comparing the alloys following rolling and post-annealing. According to the data, it shows that both the prismatic and pyramidal slip were activated, which supports the dominance of these slip systems in the alloys and an implied weakening of the basal texture.

Kernel average misorientation (KAM) maps quantify and visualize local variations in crystallographic orientation, making them useful for assessing the degree of plastic deformation and for identifying areas of local strain within grains, which has been widely applied in plasticity characterization [28,29,30,31,32,33,34]. In other words, KAM is a measure of grain spread derived from EBSD data. On the other hand, grain orientation spread (GOS) is used to average the misorientation angle for each grain separately by taking the average of the misorientation angles for each grain from EBSD data. The GOS can be used to analyze the primary strain of grains and reveal the most deformed grains. A low GOS value indicates that the crystal orientation within the grains is relatively uniform and that the recrystallization degree is relatively high [19,34]. Both KAM and GOS provide quantitative insight into lattice distortion and recrystallization state [19,34]. This is also demonstrated with a decrease in the KAM and GOS data after post-annealing as seen in Figure 4 and Figure 5, respectively. This reduces the anisotropy of the alloy, which is crucial for improved stretch formability.

Previous studies on related Mg alloys, such as AZ80, suggest that Zn addition provides deeper insight into both electronic and phononic contributions to mechanical behavior [35]. The addition of Zn promotes stronger electronic hybridization, as evidenced by increased overlap in the electronic states of Mg, Al, and Zn. This modified electronic structure is inferred to influence alloy properties and contribute to enhanced solid-solution strengthening. Consequently, the combined addition of Al and Zn is proposed to improve electronic hybridization, leading to simultaneous enhancements in strength and ductility of the AX80 alloy.

The microstructure of the AXMZ alloy after rolling and post-annealing at 450 °C for 40 min in Figure 3 reveals a pronounced bimodal grain size distribution, consisting of coarse, equiaxed grains embedded within regions of significantly finer grains. The coarse grains, with diameters on the order of tens of micrometers, exhibit smooth and well-defined boundaries with little internal contrast, indicating that they have undergone complete static recrystallization followed by grain growth and are also due to recovery. In contrast, the fine-grained regions appear clustered and irregular, suggesting spatially heterogeneous recrystallization behavior. The corresponding phase map shows that this heterogeneity is strongly correlated with the distribution of secondary phases in Figure 3b. Specifically, regions containing higher densities of intermetallic particles—comprising Ca-, Mn-, and Zn-rich phases as indicated in the phase legend—are associated with the fine-grained microstructure. These particles, particularly when present as coarse clusters, likely promote particle-stimulated nucleation (PSN), leading to localized formation of fine recrystallized grains [19]. Additionally, a dispersion of fine precipitates is observed decorating grain boundaries, especially surrounding the larger grains. These finely distributed particles contribute to Zener pinning, which locally impedes grain boundary migration. However, the relatively lower precipitate density within the interiors of coarse grains and in particle-depleted regions permit continued boundary motion, facilitating grain coarsening. Overall, the observed bimodal structure reflects the interplay between recrystallization, grain growth, and particle effects, where spatial variations in second-phase distribution govern both nucleation and growth kinetics during annealing.

In Figure 6, the TEM images reveal two dominant second-phase morphologies including rod-like or needle-like precipitates as well as fine-roughly equiaxed nanoscale precipitates. The rods appear to suggest more contrast than the matrix. From the rolling, there appears to be dislocation networks with heterogenous nucleation sites. The rolling also shows the rod-like features to be relatively short and thick with the surrounding region showing non-uniform matrix contrast with a high dislocation density. From the annealing at 450 °C and 40 min, the images show grain coarsening with some spheroidization of the finer particles. There also appears to be obvious growth and coarsening of the rod-like precipitates as well as a reduction in the matrix dislocation density. These features are consistent with transition/metastable precipitates evolving toward equilibrium rod-like phases. This description is also consistent with well-established works [36,37,38].

In reference to Figure 7, the various precipitates are observed with and without the addition of Zn. With the AXM alloy, adding Ca to increase the Ca/Al ratio encourages the formation of brittle fine lamellar (Mg_2_Ca) precipitates, which can lead to a more brittle fracture mode. Also, Ca-rich precipitates consisting of Al_2_Ca and Mg_2_Ca tend to sit at the grain boundaries, which enhances the strength and creep resistance but also reduces ductility where these high-density secondary phases act as fracture initiators [25]. In contrast to the rolled alloy, post-heat-treatment of AXM has shown that the Al_2_Ca (metastable precipitate) led to improved strength and ductility [9]. Other fracture features have typically included dimpled rupture characteristics with fin, equiaxed dimples indicating microvoid coalescence from Al_2_Ca particles [25]. The addition of Zn to AXM alloys creating AXMZ has shown to increase the ductility and improve the formability by modifying the texture from basal to quadruple basal texture [19,20,24]. With the addition of Zn, the alloy tends to exhibit a change from shear cleavage to a mixture of cleavage and finer, deep dimples with a higher Zn content, indicating an enhanced ductile fracture behavior. Some of the key impacts of adding Zn include enhanced elongation due to increased grain refinement and altered texture with the presence of coarse secondary particles.

Zn addition has been shown to increase strength through a combination of solid solution strengthening and fine-particle precipitation hardening [19]. For example, adding 0.6 wt% Zn to Mg-2Al-0.5Ca has been shown to increase the ultimate tensile strength (UTS) to over 300 MPa. In the AXMZ alloys, strength and ductility were improved after adding Zn as seen in Figure 8. In terms of ductility and formability, the combination of refined grain structure (fine, homogenous grains) and weak texture allows for enhanced dislocation slip over twinning, significantly increasing elongation (beyond 20% in the AXMZ205041 alloy) and improving sheet stretch formability. While low levels (0.5 to 1.0 wt%) enhance both strength and ductility, excessive Zn has been shown to lead to excessive coarse precipitates, potentially restricting ductility in some cases [35]. Thus, there is a sweet spot regarding the range of Zn added to improve properties, specifically ductility. Reducing the strong basal texture was demonstrated to be a clear spreading and weakening of the basal texture after post-annealing for the single-pass DSR specimens, as compared to the DSR condition, as seen in Figure 2. This evidence of basal texture weakening demonstrates that Zn is also an effective contributor to ductility.

Strain hardening in AXM alloys is increased by adding Zn by increasing the solute concentration in the alpha-Mg matrix, which strengthens the alloy through solid solution strengthening and slower dislocation motion. In addition, dislocation density and interactions of dislocations and grain boundaries with precipitates also contribute to the strain hardening of the material. Furthermore, Zn addition often alters the texture from a sharp basal texture to a weaker one, which can delay necking and increase the strain-hardening exponent that also can contribute to strengthening of the alloy [39]. Some of the microstructural mechanisms behind this texture weakening include a solute drag effect where the zinc and calcium atoms segregate to the grain boundaries which reduce boundary energy and mobility causing the pinning effect that suppresses abnormal grain growth during dynamic recrystallization [5,40]. In addition, because the grains are forced into a more randomized orientation, the critical resolved shear stress (CRSS) ratio between the basal and non-basal slips is effectively lowered. This facilitates the activation of a pyramidal slip and tensile twinning [5,40,41,42,43]. The solute Zn also encourages a completely recrystallized ultrafine, and homogenous structure, wherein the finer grains distribute stress more evenly, delaying crack initiation and improving overall uniform elongation [5,40,41,42]. The co-segregation of Zn and Ca strengthens the grain boundaries, making the material more resistant to intergranular fracture even when subjected to intense plastic strain during processing like bending or stretch forming [5,40]. This results in improvement in mechanical performance and formability as observed in the improved strength of the AXMZ alloys as observed in Figure 8.

## 5. Conclusions

Some of the most important findings in this study show a comparison of the AXM and AXMZ alloys and their role in formability as follows:

1. Single-pass DSR significantly refined the grain structure of the AXM alloy and increased strength but reduced ductility due to strong basal texture and large lattice distortion.
2. Post-annealing effectively activated static recrystallization, with near-complete SRX achieved at 450 °C for 40 min. This same post-annealing time and duration was applied to the AXM and AXMZ alloys, which demonstrated improvement in ductility while retaining strength.
3. Texture weakening, reduced lattice distortion, and formation of strain-free grains led to a substantial improvement in ductility, reaching ~12% in the base AXM alloy, while ductility was significantly improved to beyond 20% in the AXMZ alloys.
4. Optimization occurred through post-annealing, which improved ductility/formability but also preserved tensile strength above 200 MPa.
5. The DSR plus post-annealing route represents a viable processing strategy for high-strength, formable Mg alloy sheet products.
6. The strength of the AMX alloy was enhanced by the addition of Zn through solid solution and fine second-phase precipitates that reduce the mobility of dislocations while the ductility was improved by weakening of the basal texture.

The key finding of this study is that multi-step differential speed rolling, combined with post-annealing and targeted alloying, can effectively optimize the balance between strength and ductility in Mg-based alloys, thereby enhancing their formability. Although post-annealing reduces strength through the static recrystallization (SRX) process, the resulting strength remains significantly higher than that of the initial T4 condition. This retained strength, coupled with improved ductility, enables enhanced room-temperature formability. Overall, this optimization strategy is critical for developing materials that maintain mechanical integrity while offering superior shaping capability in manufacturing applications.

## Figures and Tables

**Table 1:** EBSD data for the AXM and AXMZ alloys.

Specimen	Grain Size (mm)	Pole Intensity (MUD)
AXM20504, T4 (Base alloy)	60.3 ± 54.3	6.13
AXM20504, DSR 4:2 at 40%	31.1 ± 14.3	9.82
AXM20504, DSR & Annealed 450 °C for 40 min	32.2 ± 8.93	4.22
AXMZ2050405, DSR 4:2 at 40%	26.9 ± 13.8	9.30
AXMZ2050405, DSR & Annealed 450 °C for 40 min	30.6 ± 12.0	3.22
AXMZ205041, DSR 4:2 at 40%	29.4± 13.5	7.71
AXMZ205041, DSR & Annealed 450 °C for 40 min	31.0 ± 12.3	2.50

**Table 2:** KAM and GOS data for the AXM and AXMZ alloys.

Alloy Process Condition	KAM	GOS
AXM20504 (Rolled)	1.74 ± 1.23	6.54 ± 3.75
AXM20504 (Annealed)	0.35 ± 0.30	0.43 ± 0.40
AXMZ2050405 (Rolled)	1.65 ± 1.54	2.86 ± 2.80
AXMZ2050405 (Annealed)	0.27 ± 0.25	0.45 ± 0.43
AXMZ205041 (Rolled)	0.47 ± 0.43	0.92 ± 0.90
AXMZ205041 (Annealed)	0.26 ± 0.25	0.47 ± 0.45

**Table 3:** Mechanical property data for AXM and AXMZ alloys.

Specimen	Yield Strength (YS) MPa	Ultimate Tensile Strength (UTS) MPa	Elongation (E) mm/mm
T4	125.0 ± 6.3	160.0 ± 8.9	5.8 ± 0.9
AXM20504 DSR 4:2, 40%	150.2 ± 6.6	265.0 ± 13.3	4.4 ± 0.7
AXM20405, DSR 40% & Annealed 450 °C, 40 min	120.7 ± 6.1	226.0 ± 11.3	12.8 ± 1.9
AXMZ2050405 DSR 4:2, 40%	125.0 ± 6.5	260.0 ± 10.1	6.5 ± 0.9
AXMZ2050405 DSR 4:2, 40% & Annealed 450 °C, 40 min	112.0 ± 5.6	201.0 ± 11.9	14.0 ± 2.1
AXMZ205041 DSR 4:2, 40%	220.0 ± 6.8	275.0 ± 13.6	3.5 ± 0.5
AXMZ205041 DSR 4:2, 40% & Annealed 450 °C, 40 min	123.0 ± 6.4	231.0 ± 11.5	21.3 ±2.6

**Table 4:** Schmid property data for AXM and AXMZ alloys.

Specimen	Schmid Factor (Prismatic)	Schmid Factor (Pyramidal)
T4	0.32 ± 0.06	0.35 ± 0.04
AXM20504 DSR 4:2, 40%	0.27 ± 0.07	0.38 ± 0.05
AXM20405, DSR 40% & Annealed 450 °C, 40 min	0.28 ± 0.06	0.39 ± 0.11
AXMZ2050405 DSR 4:2, 40%	0.34 ± 0.11	0.41 ± 0.08
AXMZ2050405 DSR 4:2, 40% & Annealed 450 °C, 40 min	0.39 ± 0.13	0.45 ± 0.10
AXMZ205041 DSR 4:2, 40%	0.33 ± 0.07	0.40 ± 0.04
AXMZ205041 DSR 4:2, 40% & Annealed 450 °C, 40 min	0.43 ± 0.13	0.45 ± 0.10

## Data Availability

The original contributions presented in this study are included in the article. Further inquiries can be directed to the corresponding authors.
